# Comment on Hu et al. Determination of 2-Pentanol Enantiomers via Chiral GC-MS and Its Sensory Evaluation in Baijiu. *Foods* 2022, *11*, 2584

**DOI:** 10.3390/foods12071449

**Published:** 2023-03-29

**Authors:** Rafał Frański

**Affiliations:** Faculty of Chemistry, Adam Mickiewicz University, Uniwersytetu Poznańskiego 8, 61-614 Poznań, Poland; franski@amu.edu.pl

Recently, Hu et al. have published a very interesting paper concerning the GC-MS analysis of 2-pentanol enantiomers in four types of Baijiu, a strong alcoholic beverage, which is of importance to Chinese social culture [1]. The determination of enantiomers is always a difficult task from the analytical point of view. The first approach (an indirect method) that can be used for the determination of enantiomers is to convert them into diastereomers, which then can be separated by using standard chromatographic methods on a column with a non-chiral stationary phase. The second approach (a direct method) requires the use of a column with a chiral stationary phase that allows the separation of enantiomers without converting them into diastereomers [2]. Hu et al. chose the direct method. Since enantiomer separation is often poor even on a chiral column, the authors have tested eight chiral columns and have found the one that most effectively separates 2-pentanol enantiomers and that ensures high intensities of the corresponding peaks, namely a CYCLOSIL-B capillary column [1]. Prior to the GC-MS analysis, samples were subjected to two sample pre-treatment procedures: direct injection and liquid–liquid extraction. Both procedures yielded very good linearity, low detection limits, etc., although the authors claim in conclusion that the liquid–liquid extraction was more suitable for the purpose of the work. Besides GC-MS analysis, the results of a sensory analysis performed by 10 trained judges have been also included in the paper. It is undisputable that the results obtained by the authors may be very useful for the determination of the origin of Baijiu and its production procedures, and, as a consequence, for tracing adulteration and counterfeit detection.

The only issue, which in my opinion is disputable, in the paper by Hu et al. is the ions which were chosen for the identification of the title compounds during the GC-MS analysis, identified on page four: “ …and qualitative ions (*m*/*z*) of (R)- and (S)-2-pentanol were 75, 73, and 60 and quantitative ions (*m*/*z*) were 51.” [1]. In my opinion, these ions are not characteristic ones which are formed from 2-pentanol under an electron ionization (EI) condition. The only one from the above, which is clearly seen in the EI mass spectrum of 2-pentanol, is that at *m*/*z* 73, although it is only the fourth most abundant ion, taking into account the relative intensities (ri). The ions characteristic of 2-pentanol are at *m*/*z* 45 (100% ri), 55 (~25% ri), 43 (~15% ri) and 73 (~10% ri), as can be checked in the common mass spectral databases (https://pubchem.ncbi.nlm.nih.gov/, https://massbank.eu/MassBank/, https://webbook.nist.gov/chemistry/, accessed on 1 August 2022) and in the published EI mass spectra [3,4,5]. Although some of the published data concerning the characteristic ions of 2-pentanol are a little different from the above (with respect to the ri values and/or *m*/*z* values), the base peak is always that at *m*/*z* 45 [6,7,8]. Figure 1 shows the EI mass spectrum obtained during the GC-MS analysis and the plausible fragmentation pattern of pentanol (CAS no. 6032-29-7; for experimental details, see [9]).

Therefore, it is a matter of discussion why Hu et al. chose the ions at *m*/*z* 75, 73, 60 and 51 in order to analyze 2-pentanol. Abundant ions at *m*/*z* 75 and 73 are the ones characteristic of trimethylsilyl derivatives which are often used in GC-MS analysis [10]. However, the authors did not use this derivatization procedure. The ion at *m*/*z* 51 is characteristic of the presence of some aromatic compounds, e.g., benzene, benzyl alcohol, and anisole, although it is not very abundant (https://webbook.nist.gov/chemistry/, accessed on 1 August 2022). The ion at *m*/*z* 60 is rare in the EI mass spectra of organic compound, although it is the molecular ion of acetic acid and is abundant in the EI mass spectrum of this common compound, which of course can be also present in the samples analyzed by Hu et al.

Some of the papers devoted to an GC-MS analysis of volatile organic compounds, including 2-pentanol, contain information that the compounds were identified by comparing their mass spectra with the mass spectra in the database [11,12,13,14,15], and sometimes with the similarity indexes [14,16], retention indexes [16,17,18] or exemplary ions that have been mentioned [15], whereas sometimes no data have been provided [19]. In my opinion, at least a few exemplary mass spectra should be always provided, e.g., in the Supplementary Materials.

I have two additional minor comments concerning the paper by Hu et al. [1]. On page two, the authors claim that “2-Pentanol, also known as secondary amyl alcohol, is considered to be an important volatile substance in Moutai [26], Yanghe Daqu [27], Wuliangye [28], …”. However, 2-pentanol, although detected in the cited references, has not been mentioned as being an important compound in the mentioned alcoholic beverages [16,17,18]. Two sentences in the paper seem to be awkward, which are on page one: “As shown in Table 1, chiral compounds in alcoholic beverages usually used direct injection…”, and on page three: “Take 5 µL 2-pentanol racemate with anhydrous ethanol…”.

It has to be stressed that the paper by Hu et al. is interesting and that the presented results may find practical application provided that the ions used for the GC-MS analysis of the title compounds are appropriate. For the possible repetition of the experiment performed by Hu et al., an explanation provided as a corrigendum or a reply to this comment will be greatly appreciated.

## Figures and Tables

**Figure 1:**
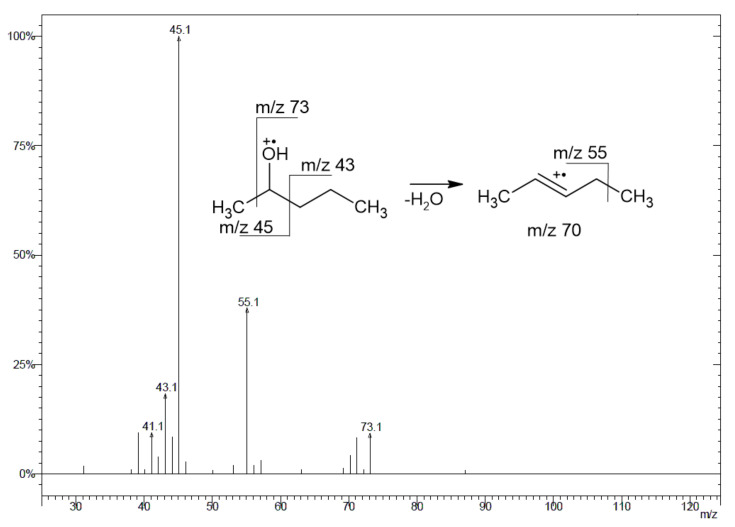
EI mass spectrum of 2-pentanol and plausible fragmentation pattern.

## Data Availability

The data presented in this study are available on request from the corresponding author.
